# Predicting the gender of individuals with tinnitus based on daily life data of the TrackYourTinnitus mHealth platform

**DOI:** 10.1038/s41598-021-96731-8

**Published:** 2021-09-15

**Authors:** Johannes Allgaier, Winfried Schlee, Berthold Langguth, Thomas Probst, Rüdiger Pryss

**Affiliations:** 1 Institute of Clinical Epidemiology and Biometry, University of Wuerzburg, Wuerzburg, Germany; 2 Department for Psychiatry and Psychotherapy, University of Regensburg, Regensburg, Germany; 3 Department for Psychotherapy and Biopsychosocial Health, Danube University Krems, Krems an der Donau , Austria

**Keywords:** Computer science, Machine learning, Signs and symptoms, Psychology

## Abstract

Tinnitus is an auditory phantom perception in the absence of an external sound stimulation. People with tinnitus often report severe constraints in their daily life. Interestingly, indications exist on gender differences between women and men both in the symptom profile as well as in the response to specific tinnitus treatments. In this paper, data of the TrackYourTinnitus platform (TYT) were analyzed to investigate whether the gender of users can be predicted. In general, the TYT mobile Health crowdsensing platform was developed to demystify the daily and momentary variations of tinnitus symptoms over time. The goal of the presented investigation is a better understanding of gender-related differences in the symptom profiles of users from TYT. Based on two questionnaires of TYT, four machine learning based classifiers were trained and analyzed. With respect to the provided daily answers, the gender of TYT users can be predicted with an accuracy of 81.7%. In this context, worries, difficulties in concentration, and irritability towards the family are the three most important characteristics for predicting the gender. Note that in contrast to existing studies on TYT, daily answers to the worst symptom question were firstly investigated in more detail. It was found that results of this question significantly contribute to the prediction of the gender of TYT users. Overall, our findings indicate gender-related differences in tinnitus and tinnitus-related symptoms. Based on evidence that gender impacts the development of tinnitus, the gathered insights can be considered relevant and justify further investigations in this direction.

## Introduction

Many people experience a long-term noise in their ears, which is widely known as tinnitus, also described as a whistling or ringing sound^1^ in the ears. About 10–15% of the worldwide population report this kind of symptoms^2,3^. Although many people perceiving tinnitus do not experience a considerable burden, about 2.4% of the worldwide population severely suffers from tinnitus on a daily basis^4^. In most of these cases, tinnitus is a subjective perception that can only be perceived by the affected person. Inversely, rare forms of tinnitus exist, for which the perceived sound is caused by a source in the body that can be objectively measured (e.g., blood flow or muscle contractions). As an important consequence of the discussed aspects, no general treatment, which is able to effectively reduce tinnitus symptoms like loudness and its related fluctuation, exists yet. On the individual basis, tinnitus can be reduced, for example, by the use of cognitive behavioral therapies^5^. To characterize the general status of available treatments with respect to the well-known heterogeneity of tinnitus patients^6,7^, they are rare and their development is difficult.

To better and more effectively deal with this heterogeneity, researchers often focus on the identification of subgroups of tinnitus patients. Identified subgroups might be used for investigations on treatments for an identified subgroup instead of a general treatment for all tinnitus patients. However, the clustering of tinnitus patients through the identification of subgroups is not an entirely new research question. Hitherto, several approaches aimed at the clustering of tinnitus patients depending on their symptom profiles^8,9^, or depending on neuroimaging data^10^. Furthermore, the authors of^11^ developed the Tinnitus Primary Function Questionnaire to examine the effect of tinnitus on thoughts and emotions, hearing, sleep, and concentration. The authors established correlations between these four effects and derived secondary limitations for the individuals in their daily life. The consideration of potential differences in gender are another approach on subgroup research. A recent special issue shows the latter kind of interest in research^12^. In the already published articles of this special issue, for example, one work deals with gender differences of chronic tinnitus patients^13^. All of the presented works show that gender differences are a valuable research direction in particular and with respect to research on subgroups of tinnitus patients in general. In addition, research evidence exists that the gender impacts the development of tinnitus and the response to treatments. For example, in this recent work^14^, the authors investigated treatments of 316 patients and found significant treatment differences between males and females. For instance, females improved better in orofacial therapies. Or, in the work of^15^, it was found, among other findings, that stress was positively correlated with tinnitus severity only in males. These and other findings clearly show that gender-related differences are relevant for investigations of tinnitus patients and their symptom profiles.

In the discussed context, the use of mobile applications to monitor health symptoms is becoming more and more popular, also denoted by mobile and digital health (mHealth). With respective mHealth solutions, the collection of data becomes easily possible, especially on a daily basis. Furthermore, data can be collected close to the user’s daily life with the goal to foster self-monitoring and eventually may support health care in clinical practice^16^. For example, the authors of^17^ monitored and investigated mental health conditions by using a mHealth solution, while the authors of^18^ showed the general potential and impact of mHealth applications. For TrackYourTinnitus (TYT), the daily use, among other reasons, enables individuals to be better deal with the variations of the tinnitus over time. On the flip side, mHealth solutions also revealed drawbacks, which are discussed by many recent works. For example, potential discrepancies of app developers and patients of mHealth apps are investigated more in-depth by^19^, while general challenges are discussed by^20^. In the discussed setting, it should always be kept in mind that a daily smartphone usage might also worsen the individual tinnitus situation as users are reminded about their problems on a frequent basis. However, research works exist that have shown that the daily use of mobile technology does not aggravate the overall health condition, see for example^21^. Despite such findings, the daily focus on a disease when using mHealth solutions should always be considered carefully.

For the identification of tinnitus subgroups, the collection of longitudinal ecologically valid data sets based on mHealth solutions has been recognized by several researchers. Technically, mobile crowdsensing techniques^22^ or Ecological Momentary Assessments^23^ are mainly utilized to gather the required data sets. For tinnitus research, these technologies have already shown that they can collect valuable data^24,25^. To identify subgroups of tinnitus patients, data sources established by the use of mHealth solutions have also revealed to be appropriate^26^. Several of these works have presented their findings on data of the TrackYourTinnitus platform (TYT), which was developed to evaluate daily symptom fluctuations of tinnitus patients. TYT comprises two mobile native (developed without using frameworks) applications (an Android and an iOS app), a website (http://www.trackyourtinnitus.org), and a server application that stores the data generated by the apps. The platform was developed by an interdisciplinary team of computer scientists, medical doctors, and psychologists. It can be freely used by interested users, the apps can be downloaded through the official app stores from Apple and Google. In essence, the following complete the following procedure: First, they have to fill out three registration questionnaires after downloading the app. After that, they decide on the number of daily notifications. Each notifications reminds the user to fill out a daily questionnaire, comprising so-called EMA questions, which aim at the momentary tinnitus situation of a user. In addition, the environmental sound level is collected through the microphone of the used smartphone when filling out the daily questionnaire. In terms of feedback, the app visualizes the gathered data and through the website, interested users can download their collected data. TYT does not offer further features. Although the platform aims at data for research and it could be assumed that this is of less interest, so far, the platform has gathered more than 100,000 daily questionnaires by more than 3000 users from all over the world. We learned that despite the fact that TYT is an open research project in the sense of a long-running observational study, two aspects are of importance for users to participate. First, the project is without any commercial interest. Second, data is collected anonymously except one reason. If users want to reset their password, they have to provide their mail address. In general, the secure handling of data collected by the use of a smartphone is an important aspect since smartphones provide a lot of opportunities to gather data that indirectly might reveal the user. For example, when GPS data is collected and the location of a user is sent to a central server. In general, works exist that have developed complex configurations with which users can control the provision of mHealth-related, see for example^27^. Interestingly, such works show that users are less interested to control much themselves, therefore it is important that a mHealth solutions tries to secure data and privacy in the best possible way by design. In the case of TYT, only questionnaire data and the environmental sound level are gathered, which might be also one reason to use it frequently by many users. To conclude, the TYT project is running since 2014 and revealed various investigation opportunities, including those, which were initially not planned^26,28^. Beyond TYT, other mHealth solutions have been developed and presented to support diagnosis and therapy of tinnitus patients^5,29,30^, which emphasizes the potential of mHealth in this context.

Moreover, the combination of mHealth and machine learning has become very popular recently. The directions followed in this context are manifold. On the one hand, considerations on sparse mHealth data are subject to research when using machine learning methods in the given context^31,32^. On the other hand, large mHealth data sets exist that are investigated by the use of machine learning methods^33^. Moreover, the development of new machine learning methods and the evaluation of existing ones is also considered presently^34,35^.

In this work, gender-related differences of TYT users are investigated, hereby based on the following thoughts: Existing insights on TYT, existing works on machine learning methods to identify subgroups of TYT users, and the amount of existing data of TYT users distributed between females and males. Further note that TYT is technically based on mobile crowdsensing techniques^36^ and utilizes Ecological Momentary Assessments (EMA) to capture ecologically valid data sets of tinnitus patients. Since 2014, the TYT mHealth platform has gathered more than 100,000 completed questionnaires from its users. With respect to the identification of subgroups, machine learning based investigations on the TYT source already exist. For example, in^37^, the differences of TYT Android and iOS users were investigated, while in^38^, entity (i.e., individual TYT users) similarity was investigated to label the future observations referring to an entity.

For the investigation at hand, two prerequisites are important: First, it must be defined which type of gender differences are addressed in this work. The authors of^12^ define the following important differences: the (1) biological classification encoded in the DNA and the (2) understanding of the respective social roles, behavior, and expressions. In this work, we refer our considerations to the latter type of difference. Second, it must be defined which gender-related aspects of TYT users shall be investigated. The answer to this question is that our goal is to predict the gender of the user of a provided daily assessment. A daily TYT assessment, in turn, is based on the filled-out daily questionnaire, which comprises 8 EMA questions (users can opt which questions they actually want to fill out; in addition, 1 question varies among users based on an answer given to the perceived worst symptom provided through one baseline questionnaire) that capture the current situation of a TYT user (see this work for a detailed explanation^39^). Note that TYT users have two options to fill out this questionnaire. The first option entails receiving up to 12 random notifications per day, which then remind users to fill out the questionnaire, while the second option allows users to determine fixed points in time to receive the notifications. Furthermore, baseline questionnaires, which must be answered when using the smartphone app for the first time, provide the information on the gender of a TYT user. Based on this information, 15 features were identified—out of the 8 daily questions—for the gender prediction task, covering aspects like stress, worries, arousal, depression, mood, or the loudness of the momentarily perceived tinnitus. A detailed explanation of the features is provided in Table 3.

Given these two prerequisites, the overall goal of the work at hand is the prediction of the gender of the user of a given daily TYT assessment based on machine learning methods. A binary classification is therefore accomplished that deals with the following detailed questions (note that for the classification task, technically, Sklearn^40^ has been used):

(i) Is it possible to learn a mapping function from *X* to *y* of TYT individuals, for which *X* are questions that the user answered daily and *y* is a binary target representing the gender of the respective TYT user?
(ii) Which machine learning model is mostly suitable for this task and has a high prediction power?
(iii) Which are the features with the highest importance to predict the gender?

It is briefly discussed whether other approaches have trained binary classifiers on mHealth related data with respect to research questions on gender-related differences. In general, works exist that have trained a binary classifier on mHealth data. For example, the authors of^41^ used such a classifier for respiration disorders of mHealth applications. Furthermore, approaches exist that investigated gender differences in the general context of mHealth solutions. However, their focus is different to the one that is investigated in this work. More specifically, other works^42,43^ investigate differences when using mHealth technologies from a general point of view. That means that they investigate whether there is a difference between men and women when addressing medical issues while using mHealth solutions. Yet, the focus of these works is different to the presented work: they start with the gender and try to establish which bias this might generate on the use of a solution. In contrast, this work starts from the data source and tries to predict the gender. Although these two perspectives address the same overall research context and are therefore intertwined, the research questions they are addressing are different. Still, to the best of the authors‘ knowledge, similar works that present a binary classifier on mHealth data with respect to results on gender-related differences do not exist yet.

## Results

In this section, the three research questions are discussed subsequently. First, it is discussed whether it is generally possible to solve the gender prediction task by using machine learning with relevant results. Next, the hyper-parameters of the chosen classifiers must be fine-tuned. Finally, by using the knowledge from Research Questions *i* and *ii*, the question must be answered, which of the features are mostly suitable to classify the gender. A summary of this section is provided in Table 1.

**Table 1:** Overview of the three Research Questions *i-iii*, the used classifiers and the results.

No.	Research question	Machine learning algorithm				Results
		SVM	Tree	RF	NN	
i	Is it generally possible to learn a mapping function from X to y where X are questions that the user answered daily and y is a binary target representing the gender of a user?	$\checkmark$	$\checkmark$	$\checkmark$	$\checkmark$	Precision on average: male: 81.5% female: 84.3%
ii	Which machine learning model is most suitable for this task and a high prediction power?	$\checkmark$	$\checkmark$	$\checkmark$	$\checkmark$	Mean accuracy on a fivefold cross validation set: Random Forest classifier (81.7%)
iii	Which are the features with the highest importance to predict the gender?			$\checkmark$		Most important features are: q8_4: Worries about the tinnitus q8_5: difficulties in following a conversation

SVM = Support Vector Machine, Tree = Decision Tree, RF = Random Forest, NN = Multilayer Perceptron Neural Network. A checkmark means that this classifier has been used to answer the research question.

### Research question i

In this study, gender is considered to be binary as there is no data for diverse tinnitus patients. Given that the target classes are uniformly distributed, random guessing for a binary classification task leads to an accuracy of 50% on average. Consequently, a mapping from *X* to *y* is adding information if the accuracy of a classifier is higher than 50%. If it is significantly higher than 50%, it must be decided based on the achieved accuracy whether it is actually relevant or useful. *X* was used as the (sub)set of features and *y* as the target for gender, with {male, female} as possible classes.

The classification task was accomplished using Python, as this is one of the most used languages for Machine Learning^40^, which enables comparisons to many other research results. Four classifiers from the scikit learn library were used for the investigations: A Support Vector Machine, a Multilayer Perceptron Neural Network, a Decision Tree, and a Random Forest. All of them were able to guess the gender with a significantly higher accuracy than 50%. These classifiers were selected as they are well known to get high accuracy scores for high dimensional classification tasks on small to middle-sized datasets^44–47^.

Note that the more features were added to the classifiers, the higher was the accuracy. For the testing set, a fivefold cross validation was used to avoid overfitting. As can be seen from Table 2, the random forest classifier had the highest prediction power in this distribution.

**Table 2:** Comparison of the four used classifiers in terms of precision per gender and F1-score.

Classifier	Precision male	Precision female	F1-score
Support Vector Machine	0.80	0.86	0.83
Decision Tree	0.81	0.80	0.81
Neural Network	0.82	0.83	0.83
Random Forest	0.83	0.88	0.85

Number of examples is denoted by *m* = 1702. Used features: {q1, q2, ..., q7, q8_5}, test size 20%. Note that the feature labels qx are further explained in Table 3.

### Research question ii

As there is no other satisfying metric such as training time or minimal false positives rates, it was decided to further investigate the classifiers accuracy.

**Table 3:** Description of the dataframe used for the machine learning approaches.

	Meaning	Scaling	Implementation	Count	Mean	Std
Question1	Did you perceive the tinnitus right now?	Binary	YesNoSwitch	80,969	0.76	0.43
Question2	How loud is the tinnitus right now?	Continuous	Slider in range (0,1)	80,969	0.46	0.3
Question3	How stressful is the tinnitus right now?	Continuous	Slider in range (0,1)	80,969	0.36	0.28
Question4	How is your mood right now?	Discrete	SAM from 0 to 1 with step size 0.125	80,969	0.56	0.21
Question5	How is your arousal right now?	Discrete	SAM from 0 to 1 with step size 0.125	80,969	0.26	0.22
Question6	Do you feel stressed right now?	Continuous	Slider in range (0,1)	80,969	0.28	0.24
Question7	How much did you concentrate on the things you are doing right now?	Continuous	Slider in range (0,1)	80,969	0.58	0.31
Question8_0	Because of the tinnitus it is hard for me to get to sleep	Binary	YesNoSwitch	7919	0.35	0.48
Question8_1	I am feeling depressed because of the tinnitus	Binary	YesNoSwitch	10,361	0.23	0.42
Question8_2	I find it harder to relax because of the tinnitus	Binary	YesNoSwitch	13,904	0.45	0.5
Question8_3	I don’t have any of these symptoms	NULL	NULL	NULL	NULL	NULL
Question8_4	I have strong worries because of the tinnitus	Binary	YesNoSwitch	10,839	0.27	0.45
Question8_5	Because of the tinnitus it is difficult to follow a conversation, a piece of music or a film	Binary	YesNoSwitch	11,877	0.33	0.47
Question8_6	Because of the tinnitus it is difficult to concentrate	Binary	YesNoSwitch	8220	0.42	0.49
Question8_7	Because of the tinnitus I am more irritable with my family, friends and colleagues	Binary	YesNoSwitch	3391	0.32	0.47
Question8_8	Because of the tinnitus I am more sensitive to environmental noises	Binary	YesNoSwitch	9179	0.09	0.29
Qender	0 = Male, 1 = female	Binary	Single Choice	80,969	0.26	0.44

Note that the count for the questions 8_0, 8_1, ..., q_8 is dependent on the number of individuals that selected this answer in the baseline questionnaire. If an individual selected *I don’t have any of these symptoms*, no follow-up question appeared, so that these values are NULL. SAM = Self Assessment Manikin^65^.

**Figure 1:**
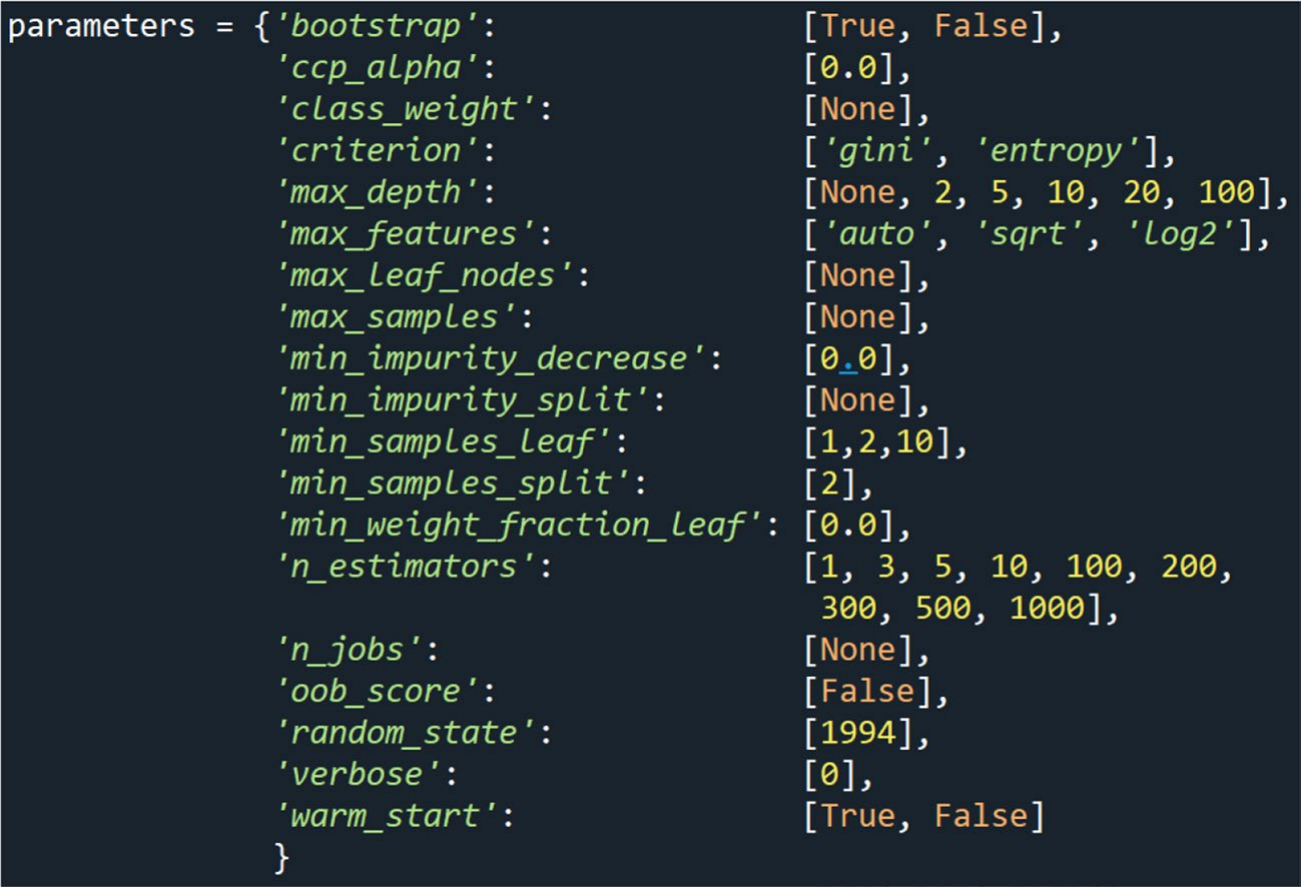
Set of hyper-parameters for a grid search in order to improve the forest’s accuracy. Note that not all hyper-parameters have be varied, such as n_jobs, oob_score or verbose. Only hyper-parameters were varied that have a higher impact on the accuracy score. However, static parameters are listed for the purpose of integrity.

To do so, a fine-tuning of the hyper-parameters of the Random Forest classifier was performed. This tuning is also known as a grid search^48,49^. Therefore, the hyper-parameters of interest were selected, which can be seen in Fig. 1. Then, one of the hyper-parameters was varied while keeping all others constant. The resulting parameters-dictionary was passed to the Random Forest classifier into the same training and testing set of the approaches of Research Question *iii*, again with a fivefold cross validation^50–52^. Here, a fivefold split was used instead of a tenfold split for the purpose of having a sufficient testing size. Additionally, this allows to speed up training and testing time as well as to vary more hyper-parameters within the grid search. The cross validation further prevents the Random Forest from overfitting of the training set^53^. For each possible combination of the parameters dictionary, the accuracy was saved. After trying all variations, the variation with the highest accuracy determined the final parameters set up of the Random Forest classifier in the testing set.

The number of decision trees in the random forest was increased up to 1,000 for a slight improvement of the overall accuracy. However, a further increase of n_estimators did not improve the score in the testing set. If the max_depth parameter was lowered to 10, the lowest standard deviation of 2% within the fivefold cross validation was attained. The best ranked Random Forest classifier received an accuracy of 87% in the first cross validation set. The average cross-validated test score is **81.65%**, with a standard deviation of 4%.

### Research question iii

There exist several techniques to determine feature importance, such as random, heuristic, or complete approaches^54^. In order to answer the third Research Question iii, three strategies were pursued. Before the strategies were accomplished, a sub-dataframe was created that contains the feature of interest and the target gender. This sub-dataframe was then filtered, so that it equally contains 50% men and 50% women.

As the first strategy, a closer look was put on the random forest approach. Importantly, it has no bias in terms of the underlying distribution of the mapping function. The forest simply measures the impact in accuracy. The higher the accuracy score for a mapping from a feature to the target is, the higher its impact on the target is. The second approach tried to measure the impact of single features using correlations with the target gender. The correlation matrix also helps the authors to get a more detailed insight into the cross-correlation between the features and a single-viewed impact of a feature on the target. The higher the correlation is, the higher the impact to the target is. Note that the correlation method varied with the scaling (binary, discrete, continuous) of a feature. For a univariate classification on gender, a rise in accuracy was expected if the correlation rises. Third, the permutation importance for a univariate Random Forest classification per feature was calculated^55^ as follows: First, the classifier was trained on a training set. Then, using cross-validation, a baseline metric was evaluated on a testing set. The permutation importance was then defined as the difference of the baseline metric with the trained feature and the baseline metric with a completely random, artificial feature.

**Figure 2:**
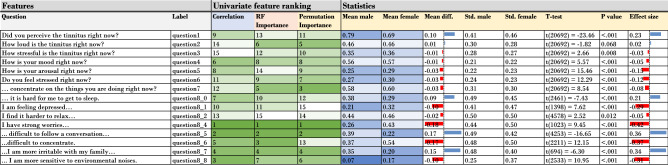
Comparison of three approaches to determine the most important feature for gender prediction. A ranking value of 1 means that this feature is most important to predict the gender.

All approaches have different units to measure the impact (Accuracy, r-value, and percentage-improvement). In order to make these three approaches comparable, a ranking of the results of the three approaches was created (see Fig. 2), and statistics for the two gender groups added, respectively. The dynamic questions q_i, with i = 0, 1, ..., 8 have on average a better ranking than the questions q_1, q_2, ..., q_7. Throughout all three approaches, *strong worries* (ranked first) and *difficulties in following a conversation* (ranked second) are the two most important features in order to predict the gender. The p-value column shows that these gender differences are all significant. From a statistical point of view, the mean difference between the two groups *male* and *female* generally supports the hypothesis that male individuals experience tinnitus differently than female individuals.

## Discussion

**Figure 3:**
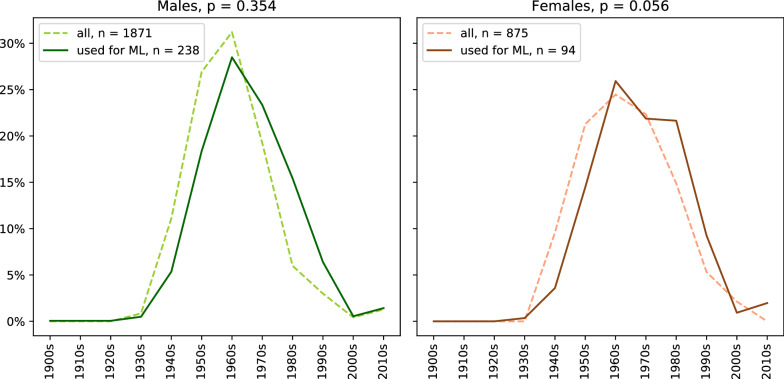
The dashed lines denote the age distribution for the all individuals, whereas the solid lines indicate the subset of individuals used for the machine learning calculations. This subset has a size of *m* = 11,877, and contains 238 + 94 individual users. For all users, *m* equals to 80,969. Note that the high p-values for both groups indicate equality of the age distribution.

**Figure 4:**
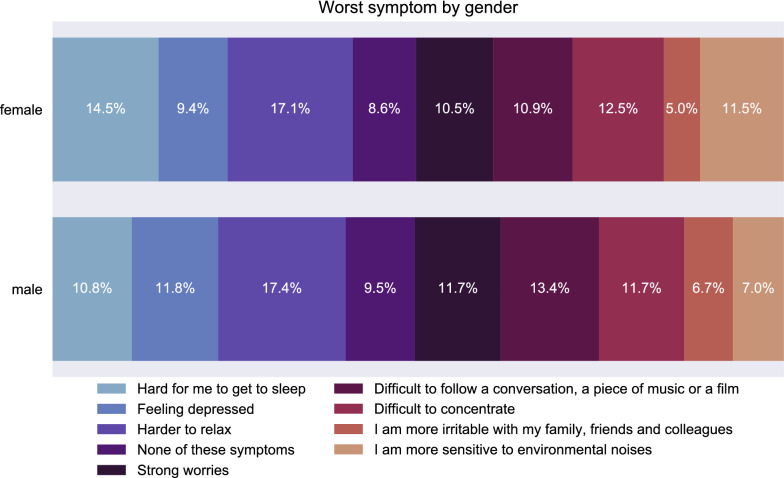
Distribution of the worst symptom grouped by gender in a horizontal stacked plot. Each row of the figure adds up to 100%.

**Figure 5:**
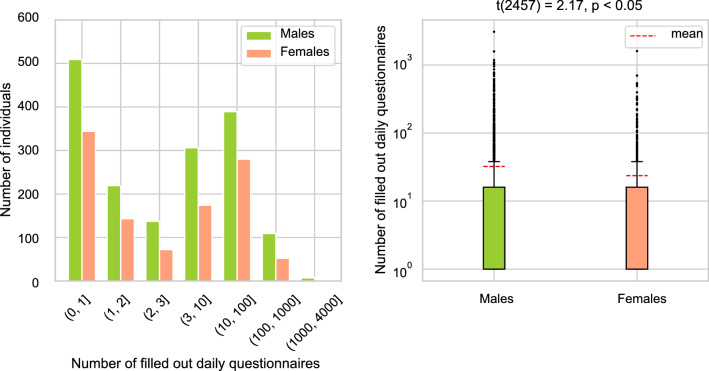
Number of filled out daily questionnaires per group (left) and per gender (right). The red-dashed line in the right plot indicates the mean value. Most of the individual users filled out the questionnaire only once. On average, men answered the questionnaire 32 times (± 124 std), and women 24 times (± 82 std) with t(2757) = 2.17 and p < 0.05. Notably, there is one male user that filled out the daily questionnaire 3073 times.

The authors are aware of the fact that by including the dynamic question q8 (The follow-up questions about the worst tinnitus symptom), only a smaller subset of TYT users could be investigated (out of all individuals), which is predestined to have a higher bias. Instead of 80,966 examples, the subsets had sizes between 3400 (4%) and 14,000 (17%) user examples. The different sizes of male and female individuals by gender can also be seen in Fig. 4. That means., if q8_5 (Difficulties in following a conversation) is chosen, it means that 10.9% of the women are included in the dataset. These subsets decrease in size again once an equal split for the target (50% men and 50% women) is performed. As a conceivable result, these subsets could not be representative anymore for the underlying distribution that has a size of *m* = 80,966. Consequently, the distribution of the chosen subset was compared with and without feature q8_5 (Difficulties in following a conversation). Note that the features q1, q2, ..., q7 were always included. For both female and male individuals, the null-hypothesis cannot be rejected, namely that these samples are drawn from the same distribution, as can be seen in Fig. 3. Grouped by gender, the distribution of the whole dataset and the sub-dataset for the features *handedness* and *family history of tinnitus complaints* was also compared. For these gender-grouped features, no significant differences between the samples could be revealed. We further compared the baseline characteristics of those individuals that only filled out the baseline characteristics and those that filled out both, baseline and follow-up questionnaires (see Table 4). These two groups also show no significant differences in distribution. In addition, the completion for the daily questionnaire differs at a gender-based level and a user-based level. More specifically, most users fill out the daily questionnaire between 1 and 10 times, while others fill it out 100 times or more. The filling-out behavior can be seen in Fig. 5. This means that some users are more represented in the training and testing set than others. However, this does not lead to a different distribution of the baseline characteristics.

**Table 4:** Baseline characteristics of the Tinnitus Sample Case History Questionnaire (TSCHQ) for all individuals that filled out at least one follow-up questionnaire.

	Characteristic					
	n	Age (std)	Right-handed	Left-handed	Both sides	Existing family history of tinnitus complaints
Male	1871	49.23 (14.40)	1345 (71%)	282 (13%)	244 (16%)	426 (23%)
Female	875	46.04 (14.72)	650 (75%)	126 (11%)	99 (14%)	235 (27%)
Total	2746	48.71 (14.89)	1994 (73%)	408 (12%)	343 (15%)	661 (24%)

Individual users that registered for this study, but did not fill out at least one follow-up questionnaire, are not considered in this table.

Less notably, the gender classification accuracy increases if q_8 (worst symptom) is added. That is due to the fact that there are gender differences in the worst symptom of a tinnitus patient. If a closer look is taken at Fig. 4, striking differences can be seen in the distribution of the worst symptom. Women tend to have more difficulties in falling asleep, whereas men tend to suffer relatively more by having difficulties in following a conversation. The authors of^56^ revealed similar symptoms of individuals in their work on tinnitus problems. Understanding speech and sleep problems were ranked as the most challenging ones without grouping by gender. The symptom sensitive to environmental noises could be biased by hyperacusis. Individuals with sensitive noise perception would tend to report higher scores here. Since hyperacusis is not assessed in the baseline questionnaire, we cannot consider it. In addition, more factors might bias the discussed symptom (e.g., if one of the parents worked in a noisy factory for a longer period of time, which is not captured by TYT) (Fig. 5).

**Figure 6:**
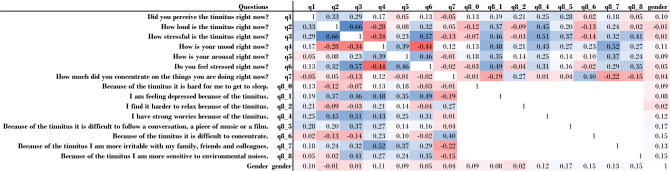
Heatmap for feature-gender cross-correlations. The last column (resp. the last row) shows the correlation of the whole data set (without equal splits for male and female individuals) with the target gender. Depending on the feature scaling, different correlation approaches (Cramer’s V, Pointbiserial and Pearson) have been used. The matrix reveals strong positive correlations between stressfulness and loudness of the tinnitus or negative correlations between mood and stressfulness of an individual user. The heatmap was formatted using MS Excel 365. Correlation metrics were calculated using SciPy 1.5.0 within a Python 3.7 environment.

When taking a closer look to the correlations of features q4 (Mood of user) and q8_7 (Depressed because of tinnitus), which is depicted in Fig. 6, a negative value can be seen. It is evident why these features should be negatively correlated. An observation with a strong positive correlation appears for the features stressfulness and loudness of the perceived tinnitus: The louder the tinnitus is, the more stressful it is.

The authors are aware of the trade-off between the depth of a tree within the forest and the standard deviation of the accuracy for a cross-validation set. A higher accuracy could be achieved for a single cross-validation set by increasing the depth of a tree. However, by increasing the depth, a higher variance must be expected between the cross-validation sets, which is an indicator for overfitting of the training set.

For Research Question iii (Which is the most important feature?), the result in the lower-ranked features is ambiguous. For the top three most important features, all three methods rank *strong worries* and *difficulties in following a conversation* firstly and secondly, respectively. For the non-changing questions q1, q2, ..., q7, however, it is not clear which one could be ranked in the middle or lower for a univariate feature importance. In summary, it can be said that the dynamic question q_8 is rated more important than the non-changing ones.

The results of the presented investigation are both clinically relevant as well as helpful for users of the TYT platform. Regarding clinical relevance, as profound indicators exist that gender differences exist for tinnitus patients^57^, TYT can be a valuable alley to learn more about daily fluctuations of tinnitus patients with respect to their gender. As our result show that the answers of the daily questionnaires can predict the gender of TYT users, inversely, the daily answers can be indicators for the symptom differences of men and women. As we further found out that the worst symptom is an important feature, we are in line with other research works beyond the scope of mHealth data^13,58–60^. Furthermore, studies that have found gender-related differences in tinnitus patients without using mHealth solutions might particularly benefit from the use of mHealth. For example, in the work presented by^61^, it is shown that gender-related differences exist for insomnia. As built-in sensors of smartphones can be used in the context of insomnia^62^, mHealth solutions might leverage findings like shown in^61^. Due to the gender-related differences we have found in TYT, it is likely that for other research questions like insomnia mHealth solutions can be helpful as well or even leverage already revealed results. We therefore conclude that in the context of gender-related differences of tinnitus patients, data that were collected with the use of mHealth solutions like TYT are relevant for medical research and clinical practice. Regarding the aspect of helping users with the findings shown here, consider, for example, the work of^58^. One outcome of the latter work describes that anxiety is only associated with bothersome tinnitus in men. Anxiety, in turn, can be easily monitored using a solution like TYT. In this particular case, the gender-related differences can be used to help, for example, men in coping with their anxiety syndrome by learning more about their daily fluctuations (if such fluctuations exist) when using TYT on a daily basis. Inversely, TYT can be used to figure out more variables that are associated with the gender and tinnitus, which might lead to the development of focused measures that may help to mitigate the tinnitus of men or woman more effectively. To conclude from a tinnitus perspective, TYT has gathered a lot of data and with this data source we were able to reveal that the question on the worst symptom (answered daily) has a high prediction power of the gender of TYT users. Since TYT asks about several worst symptoms, we consider this type of daily questions important. On the other hand, the combination with the other daily questions lead to the final result to predict the gender of TYT users, which we consider as a new outcome of TYT data and research on mHealth in this context.

Overall, the question was investigated whether the answers of male and female tinnitus patients are useful to gain a gender-based differentiation. Therefore, three research questions were investigated: (i) Is it possible to learn a mapping from *X* to *y* for the daily tinnitus questionnaire?, (ii) which is the most suitable classifier for this task, and (iii) which are the most important features? Four different classifiers of the sklearn^40^ library from Python were trained to classify the gender of a patient. The most important feature cannot be clearly determined. This result is ambiguous for different feature importance approaches. However, increasing the number of features resulted in a higher classification accuracy. Although the utilization of the possible features showed different results, the gender of the user from a provided daily questionnaire could be revealed with a relevant accuracy. The findings thus might be a valuable basis for the development of more individualized tinnitus treatments, even beyond the scope of TYT.

## Materials and methods

The study was approved by the Ethics Committee of the University Clinic of Regensburg (ethical approval No. 15-101-0204). All users read and approved the informed consent before participating in the study. The study was carried out in accordance with relevant guidelines and regulations.

### The features

For the gender prediction task, two linked data sets were used. The first one, named *Tinnitus Sample Case History Questionnaire (TSCHQ)*, is only provided to a individual *once*, and asks questions like *date of birth*, *handedness*, *family history of tinnitus complaints*, the target variable *gender*, and the worst symptom that is related with tinnitus. Baseline characteristics from this questionnaire can be seen in Table 4. Note that this table only contains individuals that filled out both, the baseline and the daily questionnaire. The worst symptom thereby can be one of the following:

- I am feeling depressed because of the tinnitus.
- I find it harder to relax because of the tinnitus.
- I have strong worries because of the tinnitus.
- Because of the tinnitus it is difficult to follow a conversation, a piece of music or a film.
- Because of the tinnitus it is hard for me to get to sleep.
- Because of the tinnitus it is difficult to concentrate.
- Because of the tinnitus I am more irritable with my family, friends and colleagues.
- Because of the tinnitus I am more sensitive to environmental noises.
- I don’t have any of these symptoms.

The second data set, named *daily questionnaire*, contains daily given answers of a registered individual. This daily questionnaire includes eight questions about the current tinnitus state, i.e., the tinnitus situation and the feelings of the individual *right now*. However, the eighth *dynamic* question depends on the worst symptom of the individual from the TSCHQ questionnaire and asks whether the individual has this specific worst symptom right now or not. If an individual user answered *I don’t have any of these symptoms* in the beginning, no question appears in the daily questionnaires. As a consequence, the number of answers for question 8 depends on the number of individuals that have selected this worst symptom in the questionnaire TSCHQ. On the other hand, the number of answers for questions one to seven equals each other. These questions are seen by every individual and are as follows:

1. Did you perceive the tinnitus right now?
2. How loud is the tinnitus right now?
3. How stressful is the tinnitus right now?
4. How is your mood right now?
5. How is your arousal right now?
6. Do you feel stressed right now?
7. How much did you concentrate on the things you are doing right now?
8. *This question depends on the worst symptom selected in the questionnaire TSCHQ.*

Depending on the features that are selected for the classification task, the number of examples *m* depends on the eighth dynamic question.

### Data preparation

The raw data set with the daily answers had the size ($m = 83349$, $n = 19$), where *m* denotes the number of samples, and *n* the number of columns. The columns of interest are individual_id, q1, q2, ..., q7, q8_1, q8_2, ..., q8_8. In total, the preparation of the data set needed much efforts, namely the following considerations and steps:

The individual_id is crucial to merge *TSCHQ* with the daily questionnaire in order to get the gender for a sample of answers. As a consequence, all rows where individual_id is NULL were dropped. This affected 1.2% of the samples, i.e., 82,351 samples remained. In the next step, values for q4(mood right now) and q5(arousal right now) were replaced that have been reported incorrectly from Android devices. For these questions, an individual user can select a position in a self-assessment manikin individual interface feature to represent his or her mood with 9 different steps (i.e., the granularity). However, the Android implementation rounds the values to tenths, which leads to incorrect values. For example, 0.13 has to become 0.125, or 0.88 has to become 0.875.

#### Missing value treatment

As every question is optional, sometimes individuals skipped questions. Therefore, the imputation module from the Sklearn library was used to fill in missing values. In order not to change the data distribution, the data set per individual was calculated. If any of the values for questions 1, 2, ..., 7 was NULL, the missing value treatment was performed. Therefore, the non-null values per column were counted. If there are two or more non-null values, an individual-specific KNN imputation for slider questions with range(0,1) and Boolean questions^63^ was performed. In case an individual user always skipped a specific question, there is no reference how this individual user usually would have answered this question. In such cases, a simple imputation was performed with a median value of the whole data set for slider questions and a most frequent replace for Boolean questions, respectively. An iterative imputation approach was not used as suggested by the authors of^64^, because then it would be required to round the estimation of Boolean questions to integer values and fit respective answers to a valid value in $\{0, 0.125, ..., 1\}$. For the dynamic variable question8, missing value treatment does not make sense, as the questions are different. For example, if an individual user has selected *feeling depressed* as a worst symptom, his or her question eight is *“Are you feeling depressed right now?”*. For all the other linked questions, the individual has never seen another dynamic question like *“Are you sensitive to environmental noises right now?”*, as the individual did not report this as the worst symptom. Consequently, these NULL values were left untreated.

#### Calculation of the correlation matrix

The values of Fig. 6 were calculated using three different methods depending on the scaling of the features. Note that it is not possible to calculate the correlations of the q8 questions to each other as they are pairwise disjoint. If both features are continuous, the Pearson correlation has been used^66^. If one feature is either discrete or binary and the other is continuous, the Pointbiserial correlation was calculated^67^. Finally, if both features are discrete or binary, the Corrected Cramer’s V correlation has been calculated^68^. Further note that Cramer’s V correlation is defined for a range of (0,1), whereas Pearson and Pointbiserial for a range of (− 1,1).

#### Univariate feature classification

For this classification task, a random forest classifier was used as proposed by the authors of^69^. In order not to get a biased estimation of the feature importance, a grouped data set per feature was calculated. As can be seen in Table 3, the number of examples *n* varies per feature. Therefore, the feature was taken with the smallest training examples (q8_7), and randomly 50% men and 50% women from the target gender were selected. In the next step, *X* was defined as the feature space of shape (*m*, *n*), with *m* = number of examples, and *n* = 1, as only one feature was used. Then, a Random Forest classifier from Sklearn was instantiated, including 80% of randomly chosen examples, which denotes the training set. Next, the accuracy on the remaining 20% of the examples was calculated, which denotes the testing set. Note that there is no development set for this subtask, as hyper-parameter tuning is not performed initially. For each feature, this procedure was repeated 10 times and the mean of those 10 accuracies were determined. The features q8_4, q8_5 (worries, difficulties in following a conversation) and q8_6 (difficulties in concentration) reach accuracy values greater than 0.58, which is significantly better than random guessing. Consequently, these features are ranked top three.

#### Comparison

Comparing the results of the three feature importance approaches, the result for the top two features is unambiguous. However, the correlation approach ranks *sensitivity on environmental noises* on a third place, whereas the permutation and random forest approach *difficulties in concentration* have different results on this rank place.

### Supervised machine learning application

#### Feature selection

After determining which variables were more and which less important for a univariate approach, the best set of features (multivariate approach) had to be identified in order to find a mapping from *X* to *y*, where *X* is a subset of all features and *y* is a binary gender prediction with male and female individuals. However, an arbitrary combination of features is only possible within the feature set of $\{q1, q2, ..., q7\}$. Only one out of the features from question 8 can be added optionally. This constraint leads to 1143 valid subsets of the data set. In order to get the best feature list, every single combination of valid subsets to a 80-20 training-testing-split of the data set was applied, before storing its accuracy and the corresponding feature list to a Python dictionary. Given a Random Forest classifier, it can be simply said that a feature list is superior to another if its accuracy on average in the testing set is higher. Without any of the dynamic questions from {q8_0, q8_1, ..., q8_8}, the best set contains the features {q2, q3, ..., q7}. Note that q1 is not included. This set leads to an accuracy of 72.7%, with a testing size of $n = 8276$. If one of the q8-questions is added to the feature set, the most promising combination contains {q1, q2, ..., q7, q8_5}, with an accuracy of 81.7% on average, and a test size of $n = 1702$.

#### Classifier comparison

**Figure 7:**
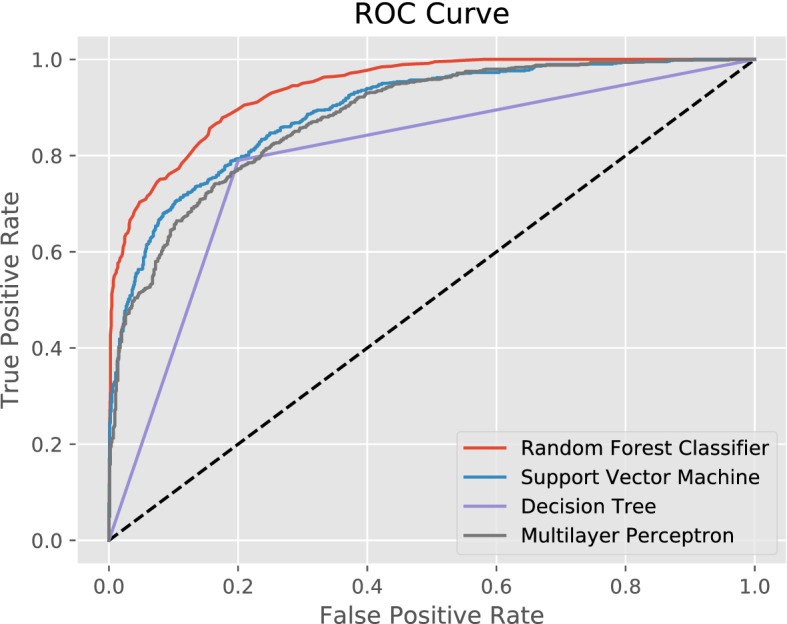
ROC curve for compared classifiers. As the decision tree contains only pure subsets, the class probabilities are either 0 or 1. This leads to a triangled ROC curve.

This section covers aspects to address Research Question *ii*: Which machine learning model is most suitable for predicting the gender of a individual user and has a high prediction power? More specifically, four supervised machine learning classifiers were investigated: A Support Vector Machine^70^, a Multilayer Perceptron Neural Network (MLP)^71^, a Decision Tree^72^ and a Random Forest^69^. With the same testing size from the previous section of $n = 1702$, the following results were obtained. The Decision Tree reached the lowest accuracy with 79%, followed by the Support Vector Machine with 80%, and the Multilayer Perceptron with 81%. The Random Forest classifier reached 86% in accuracy in the best cross validation set. The ROC curve in Fig. 7 affirms the superiority of the Random Forest classifier for this specific classification. The Support Vector Machine and the Multilayer Perceptron have a very similar performance. The Decision Tree contains only pure subsets in its final leaves, which leads to a triangled ROC curve and in this case, eventually meaning the lowest performance.

#### Hyper-parameter set-up

In a first approach, the four classifiers have been used mainly with a default set from the Python scikit-learn library^40^. Then, several hyper-parameters were slightly adjusted, i.e., the number of neurons per layer for the Multilayer Perceptron Regressor, and the splitter criterion for the Decision Tree classifier. The details of the hyper-parameters (Supplementary Information) can be seen in Listing 1.

**Listing 1:**
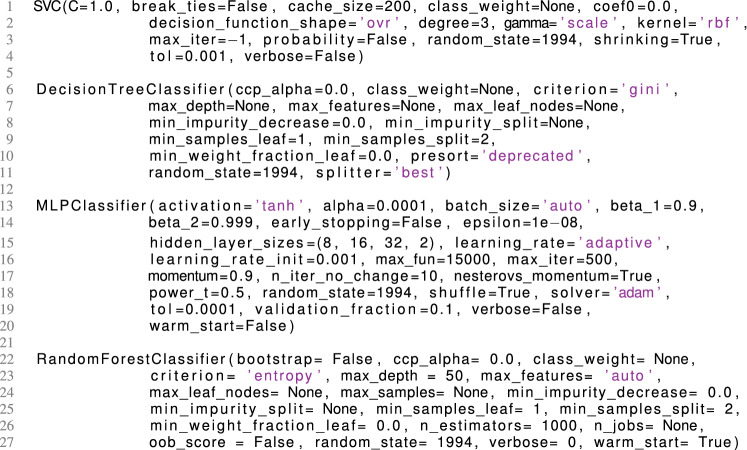
Hyperparameter set-up for the used classifiers.

According to the classifiers accuracy, the Random Forest classifier seems to be most suitable for this task, which was used to answer Research Question *ii*.

## Supplementary Information

Supplementary Information.

## Data Availability

The Python code to replicate the Machine Learning classifiers, figures and tables is available on github: https://github.com/joa24jm/tyt_gender_prediction.
