# Supplementary material for: Predicting the gender of individuals with tinnitus based on daily life data of the TrackYourTinnitus mHealth platform
Source: Sci Rep. 2021 Sep 15;11:18375. doi: 10.1038/s41598-021-96731-8 (PMC8443560; doi:10.1038/s41598-021-96731-8)
Supplement: Supplementary file 1 — Supplementary Information. [file 41598_2021_96731_MOESM1_ESM.docx]

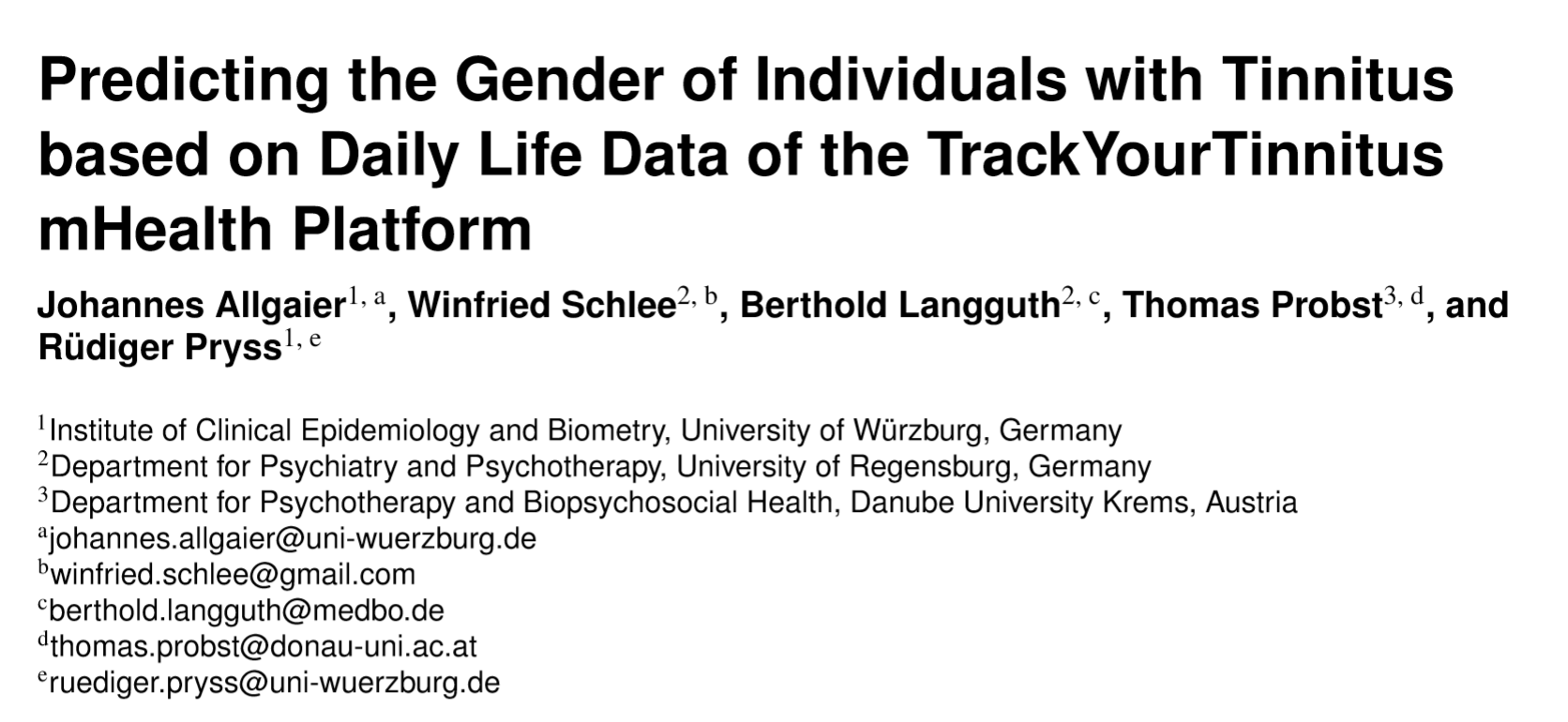


Supplementary Material

# Codebook

| **questionaire_id** | **question_id** | **question / meaning** | **implementation** | **datatype** | **answer_1** | **answer_2** |
| --- | --- | --- | --- | --- | --- | --- |
| 1 | question1 | Did you perceive the tinnitus right now? | YesNoSwitch | binary | 0 = No | 1 = Yes |
| 1 | question2 | How loud is the tinnitus right now? | Slider in range (0,1) | continous | 0 = Not audible | 1 = Maximum loudness |
| 1 | question3 | How stressful is the tinnitus right now? | Slider in range (0,1) | continous | 0 = No stress | 1 = Maximum stress |
| 1 | question4 | How is your mood right now? | Knob from 0 to 1 with stepsize 0.125 | discrete | 0 = No stress | 1 = Maximum stress |
| 1 | question5 | How is your arousal right now? | Knob from 0 to 1 with stepsize 0.125 | discrete | 0 = No arousal | 1 = Maximum arousal |
| 1 | question6 | Do you feel stressed right now? | Slider in range (0,1) | continous | 0 = Not stressed | 1 = Maximal stressed |
| 1 | question7 | How much did you concentrate on the things you are doing right now? | Slider in range (0,1) | continous | 0 = Not at all | 1 = I was fully concentrated |
| 1 | question8_0 | Because of the tinnitus it is hard for me to get to sleep. | YesNoSwitch | binary | 0 = No | 1 = Yes |
| 1 | question8_1 | I am feeling depressed because of the tinnitus. | YesNoSwitch | binary | 0 = No | 1 = Yes |
| 1 | question8_2 | I find it harder to relax because of the tinnitus. | YesNoSwitch | binary | 0 = No | 1 = Yes |
| 1 | question8_3 | I don't have any of these symptoms. | NULL | NULL | 0 = No | 1 = Yes |
| 1 | question8_4 | I have strong worries because of the tinnitus. | YesNoSwitch | binary | 0 = No | 1 = Yes |
| 1 | question8_5 | Because of the tinnitus it is difficult to follow a conversation, a piece of music or a film. | YesNoSwitch | binary | 0 = No | 1 = Yes |
| 1 | question8_6 | Because of the tinnitus it is difficult to concentrate. | YesNoSwitch | binary | 0 = No | 1 = Yes |
| 1 | question8_7 | Because of the tinnitus I am more irritable with my family, friends and colleagues. | YesNoSwitch | binary | 0 = No | 1 = Yes |
| 1 | question8_8 | Because of the tinnitus I am more sensitive to environmental noises. | YesNoSwitch | binary | 0 = No | 1 = Yes |
| 3 | 5 | Gender | SingleChoice | binary | 0 = Male, 1 = Female |  |

# Random Forest Hyperparameter Tuning for features q1, q2, …, q7 + q8_5

| **mean_fit_time** | 2.261359 |
| --- | --- |
| **std_fit_time** | 0.036827 |
| **mean_score_time** | 0.092346 |
| **std_score_time** | 0.003598 |
| **param_bootstrap** | FALSE |
| **param_ccp_alpha** | 0 |
| **param_class_weight** | None |
| **param_criterion** | entropy |
| **param_max_depth** | 50 |
| **param_max_features** | auto |
| **param_max_leaf_nodes** | None |
| **param_max_samples** | None |
| **param_min_impurity_decrease** | 0 |
| **param_min_impurity_split** | None |
| **param_min_samples_leaf** | 1 |
| **param_min_samples_split** | 2 |
| **param_min_weight_fraction_leaf** | 0 |
| **param_n_estimators** | 1000 |
| **param_n_jobs** | None |
| **param_oob_score** | FALSE |
| **param_random_state** | 1994 |
| **param_verbose** | 0 |
| **param_warm_start** | TRUE |
| **split0_test_score** | 0.870504 |
| **split1_test_score** | 0.755396 |
| **split2_test_score** | 0.81295 |
| **split3_test_score** | 0.852518 |
| **split4_test_score** | 0.791367 |
| **mean_test_score** | 0.816547 |
| **std_test_score** | 0.041453 |
| **rank_test_score** | 1 |

## Extract from classification report for best set of features

Features:

['question1', 'question2', 'question3', 'question4', 'question5', 'question6', 'question7', 'question8_5']

N = 1702


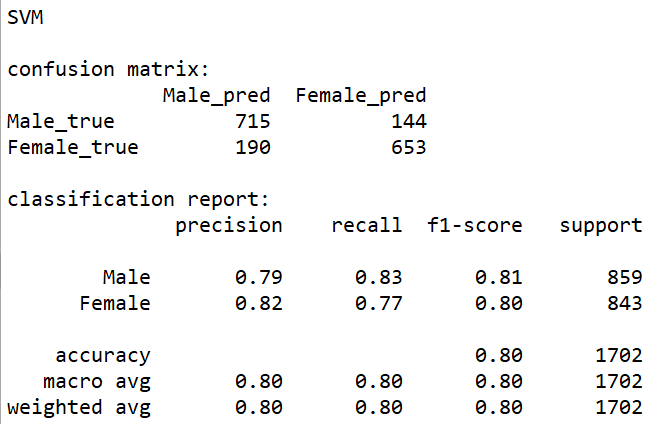

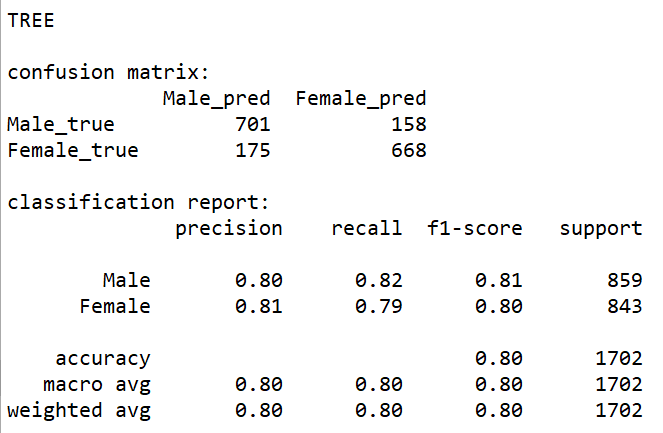


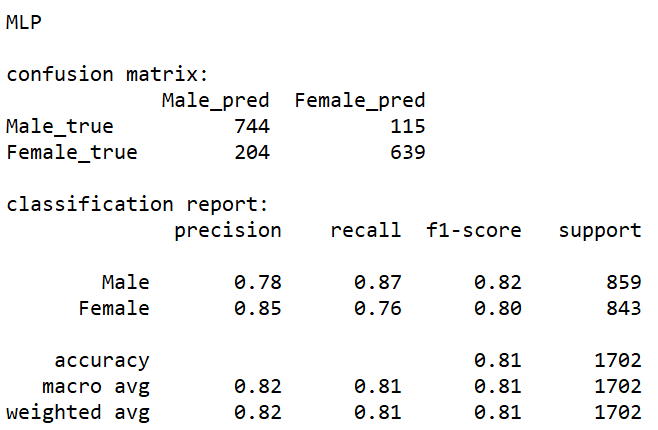

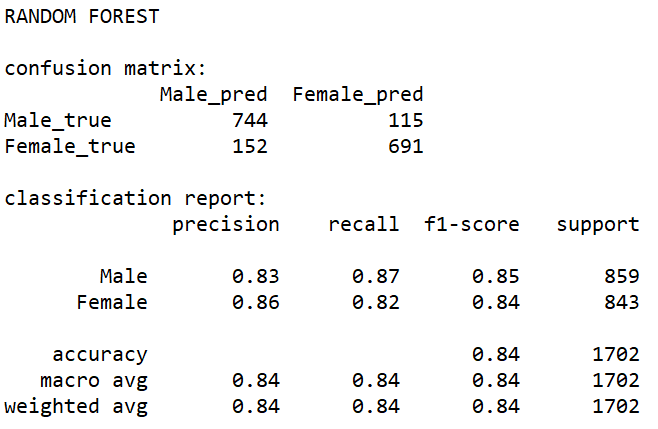


# Code can be found on github

The files on this remote repository sticks to the research questions. I.e., scripts that helped to answer research question i are the corresponding directory.

<https://github.com/joa24jm/tyt_gender_prediction.git>
